# Diagnosis and stabilisation of familial chylomicronemia syndrome in two infants presenting with hypertriglyceridemia‐induced acute pancreatitis

**DOI:** 10.1002/jmd2.12434

**Published:** 2024-06-02

**Authors:** Oliver Heath, Brooke Allender, Joel Smith, Elena Savva, Lucy Spencer, Elizabeth G. Bannister, Natasha J. Brown, Maureen S. Evans, Sharmila Kiss, Thomas H. Rozen, Joy Yaplito‐Lee

**Affiliations:** ^1^ Department of Metabolic Medicine, The Royal Children's Hospital Melbourne Australia; ^2^ Victorian Clinical Genetics Services, Murdoch Children's Research Institute Melbourne Australia; ^3^ Laboratory Services, The Royal Children's Hospital Melbourne Australia; ^4^ Department of Pathology, The Royal Melbourne Hospital Melbourne Australia; ^5^ Department of Gastroenterology University of Melbourne Melbourne Australia; ^6^ Department of Paediatrics University of Melbourne Melbourne Australia; ^7^ Paediatric Intensive Care Unit, The Royal Children's Hospital Melbourne Australia

**Keywords:** familial chylomicronemia syndrome, hypertriglyceridemia, lipoprotein lipase, pancreatitis

## Abstract

Familial chylomicronemia syndrome (FCS) is a rare disorder of triglyceride (TG) metabolism caused by loss of function variants in one of five known canonical genes involved in chylomicron lipolysis and clearance—*LPL*, *APOC2*, *APOA5*, *LMF1*, and *GPIHBP1*. Pathogenic variants in *LPL*, which encodes the hydrolytic enzyme lipoprotein lipase, account for over 80%–90% of cases. FCS may present in infancy with hypertriglyceridemia‐induced acute pancreatitis and is challenging to manage both acutely and in the long‐term. Here, we report our experience managing two unrelated infants consecutively diagnosed with hypertriglyceridemia‐induced acute pancreatitis caused by LPL deficiency. Both had elevated TGs at presentation (205 and 30 mmol/L, respectively) and molecular genetic testing confirmed each infant carried a different homozygous pathogenic variant in the LPL gene, specifically, c.987C>A (p.Tyr329Ter) and c.632C>A (p.Thr211Lys). The more severely affected infant had cutaneous xanthomata, lipemia retinalis and lipemic plasma at presentation, and required management in an intensive care setting. Acute stabilisation was achieved using insulin and heparin infusions together with the iterative implementation of a fat‐restricted diet, low in long chain triglycerides (LCT) and supplemented with medium chain triglycerides (MCT). In both cases, provision of adequate caloric intake (~110–120 kcal/kg/day) was also found to be important for a sustained TG reduction during the acute phase of management. In summary, a high index of suspicion is required to diagnose FCS in infants with hypertriglyceridemia‐induced acute pancreatitis, management of which can be challenging, highlighting the need for more evidence‐based recommendations.

**SYNOPSIS**

This paper describes the acute management of two infants with hypertriglyceridemia‐induced acute pancreatitis caused by lipoprotein lipase deficiency.

## 1 INTRODUCTION

Familial chylomicronemia syndrome (FCS) is a rare disorder of triglyceride (TG) metabolism that affects 1–2:1 000 000 individuals, often presenting in infancy and early childhood.^1^ Defects in the lipolytic cascade of chylomicrons and very low‐density lipoprotein (VLDL) result in accumulation of these circulating TG‐rich particles with ensuing elevation in fasting TG concentration (>10–100 mmol/L).^1^

Clearance of circulating chylomicrons and VLDL from the circulation normally relies on the hydrolytic activity of lipoprotein lipase (LPL) (reviewed in Laufs et al.).^2^ Several proteins interacting with LPL at the endothelial surface also affect its activity. The chaperone protein lipase maturation factor 1 (LMF1) ensures that functional LPL is secreted from adipose cells or myocytes. Glycosylphosphatidylinositol‐anchored high‐density‐lipoprotein binding protein‐1 (GPIHBP1) is necessary for transcytosis of LPL across the capillary endothelium and stabilises LPL by tethering it to the endothelium. Additionally, the apolipoprotein cofactors apo A‐V and apo C‐II enhance or stimulate lipolysis by stabilising and activating LPL, respectively.^2^

The diagnosis of FCS is best confirmed by the presence of biallelic pathogenic variants in one of five known canonical genes, with inactivating mutations in *LPL* accounting for over 80%–90% of cases.^3^, ^4^ The remaining cases involve genes encoding the aforementioned co‐factors required for LPL activation (*APOC2*, *APOA5*), maturation (*LMF1*), or binding to the capillary endothelium (*GPIHBP1*).^3^, ^4^

Clinically, the diagnosis of FCS in children can remain elusive, with incidental ascertainment of asymptomatic cases often made in the setting of a lipemic plasma sample. Non‐specific symptoms in infancy may include irritability, nausea, vomiting, and failure to thrive, while eruptive cutaneous xanthomas, hepatosplenomegaly and lipemia retinalis should also raise suspicion of the diagnosis.^5^, ^6^

Hypertriglyceridemia‐induced acute pancreatitis (HIAP) is the most severe and potentially life‐threatening complication of FCS,^7^ with TG > 20 mmol/L carrying a 5%–6% risk of mortality.^1^ Around 25% of infants with FCS can present in acute pancreatitis, while the prevalence in adulthood is higher (>85%).^4^, ^8^

Rigorous dietary intervention with a low‐fat diet is the cornerstone of long‐term treatment in FCS, although implementation is often challenging. To the best of our knowledge there are no clinical guidelines regarding the management of acute pancreatitis in infants with FCS. Here we report our experience in diagnosing and managing two consecutive infants with LPL deficiency who both presented with HIAP.

## 2 METHODS

### 2.1 Plasma lipid profile analysis

Lipid analysis was performed as described in Appendix S1. Briefly, triglycerides were measured using an enzymatic (lipase/glycerolkinase) method (reference range [RR] < 2 mmol/L) and lipoprotein electrophoresis was performed using agarose gel electrophoresis with fat red 7B staining.

### 2.2 Genomic analysis

Trio genome sequencing (tGS) for Patient 1 and trio exome sequencing (tES) for Patient 2 were performed on DNA isolated from blood using massively parallel sequencing as described in Appendix S1. Variant curation was phenotype‐driven with pre‐curated gene lists (https://panelapp.agha.umccr.org/) and classification based on ACMG guidelines.^9^

## 3 CASES

### 3.1 Patient 1: presentation

This girl is the only‐child to consanguineous healthy parents from Pakistan, with uneventful antenatal and perinatal histories. She was born at term and had symmetrical growth parameters (50th–65th centiles). There were no growth concerns in the first 6 weeks of life, but her trajectory subsequently faltered despite tolerating adequate volumes of breast/formula feeds.

**FIGURE 1:**
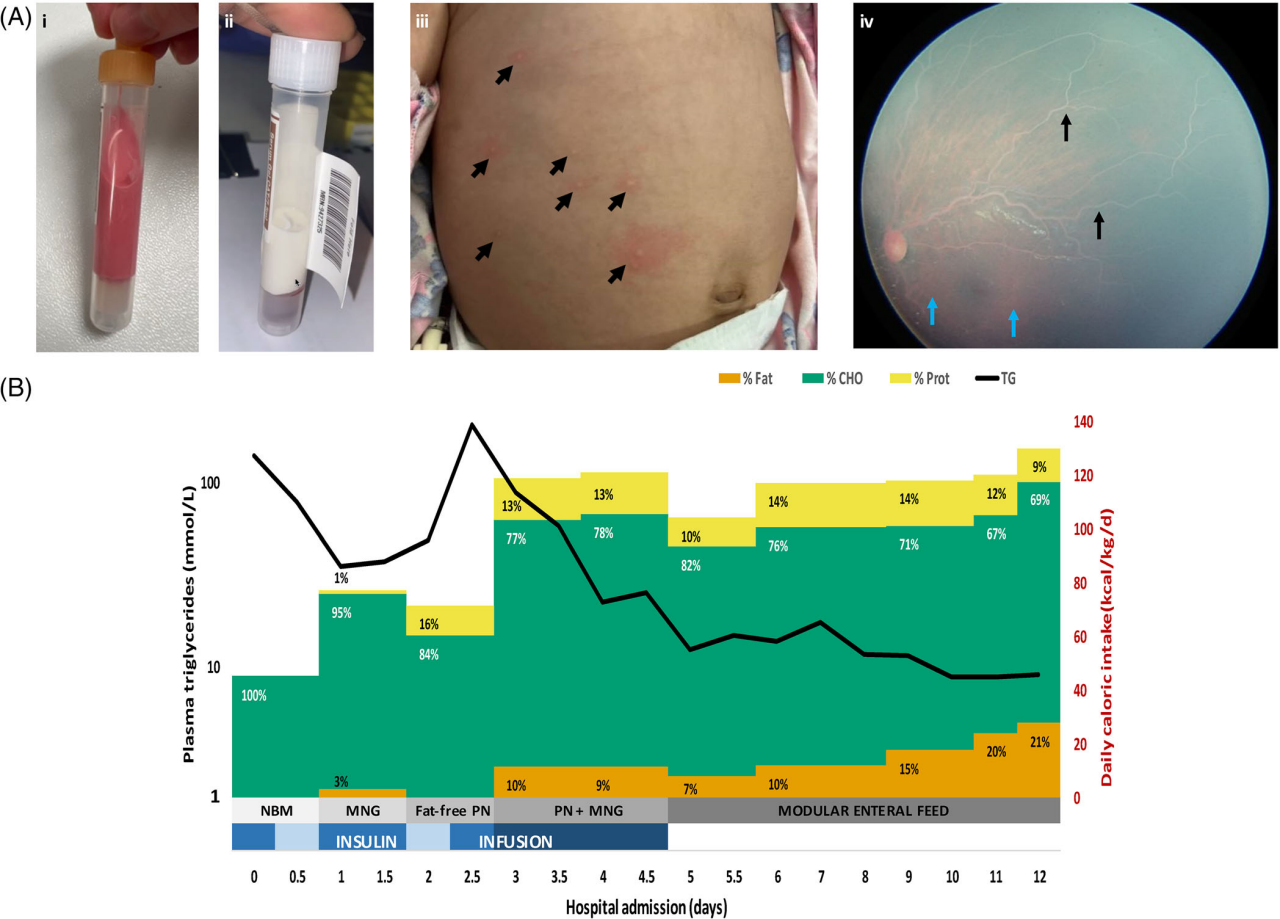
Clinical features and response to treatment in Patient 1. (A) Characteristic features of FCS: (i, ii**)** lipemic plasma (pre‐ and post‐centrifugation), (iii) eruptive cutaneous xanthomas (arrows), (iv) fundoscopy showing salmon pink retina (blue arrows) and white retinal vessels (black arrows) suggestive of lipemia retinalis. (B) Impact of diet composition on triglyceride levels. The trend in plasma TG is plotted in relation to the patient's daily caloric intake over the course of admission (d0–12). The duration of insulin treatment is also shown (blue bars); 12 hourly average infusion rates ranged between 0.01 and 0.06 U/kg/h (blue bar gradient). The content of fat (orange), carbohydrate (green), and protein (yellow) in the diet is expressed as a proportion of the daily caloric intake. Five phases of dietary intervention are shown (grey bar gradient). See text for details. CHO, carbohydrate; MNG, Monogen®; NBM, nil by mouth; PN, parenteral nutrition; Prot, protein; TG, triglycerides.

She presented at 10 weeks of age with fever, vomiting, and failure to thrive, below the third centiles for weight and length. Venous sampling showed pink lipemic blood (Figure 1A,i,ii), prompting a referral to the metabolic service for a suspected congenital disorder of lipid metabolism.

Clinical examination demonstrated growth parameters of 3.8 kg (*Z* = −3.0) for weight and 54.5 cm (*Z* = −2.0) for length. A systolic murmur was auscultated along the left sternal edge, femoral pulses were normal. Her liver edge was palpable 2 cm below the costal margin. Eruptive xanthomas were apparent on her torso (Figure 1A,iii) and she demonstrated generalised pallor. She was eumorphic with normal body fat distribution. Ophthalmological examination revealed milky‐white retinal blood vessels suggestive of lipemia retinalis (Figure 1A,iv).

Her lipid profile on admission revealed significantly elevated TGs to 140 mmol/L, total cholesterol 10.8 mmol/L (RR 2.3–4.9 mmol/L), and HDL cholesterol 0.31 mmol/L (RR 1–3 mmol/L). Lipid electrophoresis showed a streak of chylomicrons. Her lipase was raised at 1274 U/L (RR < 204 U/L), consistent with acute pancreatitis. She was hypoalbuminemic at 14 g/L (RR 29–45 g/L) and had marked anaemia with poikilocytosis (haemoglobin 47 g/L) for which she required blood and albumin transfusions. Transaminases and markers of haemolysis were all normal. Ultra‐rapid tGS identified a previously reported homozygous pathogenic nonsense variant in *LPL* (NM_000237.3): c.987C>A (p.Tyr329Ter), confirming the diagnosis of LPL deficiency (Table 1). Both parents were confirmed carriers of this variant.

**TABLE 1:** Demographics and clinical findings at presentation.

	Patient 1	Patient 2
Gender	Female	Male
Ethnicity	Pakistan	Pakistan
Consanguinity	Yes	Yes
Genetics (zygosity, ACMG classification)	*LPL*: c.987C>A (p.Tyr329Ter); homozygous, pathogenic	*LPL*: c.632C>A (p.Thr211Lys); homozygous, likely pathogenic
Age at presentation, days (months)	74 (2.5)	63 (2.1)
Initial presentation	Fever, vomiting, failure to thrive	Fever, vomiting, irritability and abdominal distension
Eruptive xanthomas	Yes	No
Lipemia retinalis	Yes	No
Peak TG, mmol/L (RR: 0.9–2.0 mmol/L)	205	30
Peak lipase, mmol/L (RR: <204 U/L)	2636	730
Haemoglobin, g/L (RR: 105–135 g/L)	42	70
Abdominal ultrasound	Bulky pancreas, with increased echogenicity	Mild splenomegaly. Peripancreatic fluid collections

Abbreviation: RR, reference range.

#### 3.1.1 Management

The patient was admitted to intensive care for management of hypertriglyceridemia‐induced acute pancreatitis and the associated risk of hyperviscosity syndrome. Dietary interventions are depicted in Figure 1B. Enteral feeding was suspended for the first 24 h and intravenous hydration commenced. Infusions of insulin (0.05 U/kg/h) and heparin were run concurrently. TG and lipase levels initially improved, decreasing to 35 mmol/L and 372 U/L, respectively. Upon re‐establishment of enteral nutrition, breastfeeding was ceased and replaced with Monogen®, a milk protein‐based formula containing medium chain triglycerides (MCT) and essential fatty acids (EFA). TG and lipase levels rebounded, however, as a result of which Monogen® was paused (day 2–3) and replaced with fat‐free parenteral nutrition (N1 solution: amino acids 25 g/L and glucose 100 g/L), providing 70–80 kcal/kg/day. Despite this, TGs and lipase continued to rise, peaking at 205 mmol/L and 2636 U/L, respectively. Over the next 48 h (day 3–5), trophic enteral feeds with Monogen® were recommenced and the parenteral nutrition graded‐up (N2 solution: amino acids 30 g/L and glucose 125 g/L). This provided a daily caloric intake of 110–120 kcal/kg/d, with the contribution from fat initially limited to 10% of total calories. Furthermore, the insulin infusion rate was also increased to 0.06 U/kg/h. These measures led to a significant drop in TGs to 12 mmol/L, a trend that was sustained (day 5–12) with transition to on‐demand enteral feeds consisting of a non‐standard formula containing Monogen®, Polyjoule®, Beneprotein®, and Seravit®. Daily caloric intake was maintained at 110–120 kcal/kg/day (103%–112% EER) to promote anabolism, while the total energy from fat was gradually increased from 10% to 20% (Table 2).

**TABLE 2:** Patient diet composition.

Patient 1						
Dietary assessment	Admission day 0	Admission day 4	Admission day 8	Admission day 12 (discharge)	First outpatient review	Latest outpatient review
Age	10 weeks 5 days	11 weeks 3 days	11 week 6 days	12 weeks 2 days	5 months	12 months
Triglycerides, mmol/L	140	45.1	11.7	8.8	4.8	7.8
Lipase, mmol/L	1274	360	23	n/a	319	1972 (asymptomatic)
Caloric requirement (EER), kcal/kg	107	107	107	107	82	80
Energy intake (EI), kcal/kg	100	131.5	120	128	96	96
% EER met	93	123	112	120	117	120
Fat, % EI	54.2	5	15	22	26	39
MCT fat, % EI	3.9	3.6	12.6	18	22	36
LCT fat, % EI	54	1.4	2	3.6	4	3.3
EFA fat, % EI	8.0	0.2	1.2	1.7	2.3	0.9
Protein, % EI	6.3	15	14	10	12	11
CHO, % EI	44	82	70	70	62.7	49
Recipe	Demand feeding mixed breast/standard infant formula	Monogen® 5.6%, Polyjoule 9.6%, Seravit® 0.8%, Beneprotein® 2.2%, KeyOmega® 0.6%, PN down‐titrating	Monogen® 9.6%, Polyjoule® 6.3%, Seravit® 0.8%, Beneprotein® 1.65%	Monogen® 15%, Polyjoule® 4%	Monogen® 16.8%, tastes of low fat solids	Monogen® 16.8%, MCT oil, low fat solids

Patient 2						
Dietary assessment	Admission day 0	Admission day 4	Admission day 5	Admission day 7 (discharge)	First outpatient review	Latest outpatient review
Age	9 weeks 0 days	9 weeks 3 days	9 weeks 4 days	9 weeks 6 days	13 weeks	6 months
Triglycerides, mmol/L	15	30.4	8.1	4	4.7	n/a
Lipase, mmol/L	730	61	41	37	n/a	n/a
Caloric requirement (EER), kcal/kg	104	104	104	102	102	82
Energy intake (EI), kcal/kg	99	38	97.9	116	102	108
% EER met	95	36	94	114	100	132
Fat, % EI	48.1	48.1	19.1	26	26	26
MCT fat, % EI	3.6	3.6	16	22	22	22
LCT fat, % EI	48.1	48.1	3.1	4	4	4
EFA fat, % EI	9.2	9.2	1.7	2.3	2.3	2.3
Protein, % EI	7.8	7.8	8.5	12	12	12
CHO, % EI	44.1	44.1	72.6	62.7	62.7	62.7
Recipe	Demand feeding standard infant formula	Standard infant formula at <50% TFI	Monogen® 11.2%, Polyjoule® 5%	Monogen® 16.8%	Monogen® 16.8%	Monogen® 16.8%, tastes of low fat solids

Abbreviations: CHO, carbohydrate; EER, estimated energy requirement; EFA, essential fatty acids; LCT, long chain triglycerides; MCT, medium chain triglycerides; n/a, not available; PN, parenteral nutrition; TFI, total fluid intake.

Patient 1 was discharged, age 12 weeks, on a non‐standard formula modular feed that provided 22% energy from fat (18% from MCT, 3.4% from LCT) and 128 kcal/kg/day (120% EER). On review at 5 months, lipase had normalised and TGs were <5 mmol/L. She transitioned onto an LCT‐restricted/MCT‐enriched solid diet, and at 12 months old, TGs remained <10 mmol/L (Table 2).

### 3.2 Patient 2: presentation

This boy is the third child to consanguineous parents from Pakistan with uneventful antenatal and perinatal histories. At 9 weeks old, he presented with fevers, vomiting, and irritability. There were no growth or developmental concerns. Clinical examination demonstrated growth parameters of 4.8 kg (12th centile) for weight and 62.5 cm (66th centile) for length. He was eumorphic and had a distended abdomen. Abdominal ultrasound showed mild splenomegaly and peripancreatic fluid collections suggestive of pancreatitis. Bloodwork unmasked elevated pancreatic lipase of 730 U/L with concomitant hypertriglyceridemia of 15 mmol/L, total cholesterol of 4.0 mmol/L (RR 2.3–4.9 mmol/L), and HDL cholesterol 0.42 mmol/L (RR 1–3 mmol/L). He had a normocytic anaemia (Hb 70 g/L, MCV 83 fL). A throat swab was positive for enterovirus RNA.

#### 3.2.1 Management

TGs normalised with 48 h of intravenous fluid management. Re‐establishment of enteral nutrition on day 2, however, caused a rise in TGs to 30 mmol/L, prompting referral to the metabolic service. The diagnosis of LPL deficiency was confirmed by rapid tES identifying a novel homozygous likely pathogenic variant in *LPL* (NM_000237.2): c.632C>A (p.Thr211Lys), and both parents were conformed carriers (Table 1).

He was commenced on a non‐standard formula containing Monogen® and Polyjoule® and providing a daily caloric intake of 95–110 kcal/kg/day (94%–114% EER), with total fat initially limited to 20% total energy. He was transitioned onto Monogen® at discharge (22% energy from MCT, 4% LCT). Review at 5 months old showed age‐appropriate weight gain and linear growth, with stable TGs <5 mmol/L and no recurrence of pancreatitis (Table 2).

## 4 DISCUSSION

We describe two infants with newly diagnosed familial chylomicronemia syndrome (FCS) who presented with acute pancreatitis.

### 4.1 Differential diagnosis of paediatric hypertriglyceridemia

Parental consanguinity and infantile onset of significant hypertriglyceridemia (>10 mmol/L) led to suspicions of an underlying genetic condition, with FCS considered most likely. A congenital lipodystrophy syndrome was deemed less likely in either case, given a normal fat distribution and lack of syndromic features. Hypertriglyceridemia can also feature in hepatic glycogen storage disorders, although hypoglycaemia with short fasting tolerance and hepatomegaly (except in GSD 0) tend to predominate the phenotype. Other differential diagnoses for genetic and acquired forms of paediatric hypertriglyceridemia include multifactorial chylomicronemia syndrome and combined dyslipidemia, both of which usually present later in childhood or adolescence and are associated with abnormalities in other lipoproteins and generally less severe hypertriglyceridemia.^10^

### 4.2 Phenotype–genotype correlation in LPL deficiency

There are no biochemical markers that distinguish the different causes of FCS, although LPL deficiency is the most prevalent.^4^ Genetic testing is required to confirm the diagnosis, with the mutational spectrum of the gene including frameshift, nonsense, splice, and missense variants. Most reported pathogenic/likely pathogenic variants are predicted to cause loss of function, although this has not been demonstrated for all, including our novel missense variant. No clear genotype–phenotype correlation has been established to date.^11^, ^12^ The Y329* in Patient 1 is predicted to cause nonsense‐mediated decay and loss of protein. The Thr211Lys in Patient 2 involves exon 5, consistent with previous suggestions that most of the deleterious missense variants in *LPL* involve exons 5 and 6 and cause LPL deficiency through LPL protein homo‐dimer instability.^13^, ^14^

### 4.3 Pathophysiology of clinical features in FCS

Clinical features in FCS can be heterogeneous. Fasting hyperchylomicronemia, can result in distinctive lipemic plasma samples that are often incidentally detected. Eruptive xanthomas and hepatosplenomegaly may occur because of triglyceride uptake by macrophages in the liver, spleen, and skin, while the characteristic appearance of lipemia retinalis results from light scattering by chylomicrons in retinal veins.^15^

Severe hyperlipidemia often hampers initial investigations, making full blood count and coagulation profile parameters unreliable. Our two patients were anaemic on presentation, with no features of haemolysis or of another aetiology. A case series describing 16 infants with LPL deficiency also reported normocytic anaemia of unclear aetiology in 44% of patients, which resolved with correction of their hypertriglyceridemia.^5^ It is possible that lipid infiltration into the marrow contributed to the anaemia in these patients.

Hypertriglyceridemia‐induced acute pancreatitis (HIAP) is the most severe complication of FCS,^16^ and can potentially lead to persistent organ failure and death.^17^ Plasma hyperviscosity and aberrant chylomicron hydrolysis in the pancreatic capillaries releases free fatty acids that damage the vascular endothelium and activate trypsinogen, with pancreatic autodigestion ensuing.^18^ The risk of HIAP decreases with reduction of TGs to <5 mmol/L.^7^

### 4.4 Management considerations in FCS‐related HIAP

Identifying FCS as the cause of HIAP is important for patients to receive proper care, although to the best of our knowledge, there are no comprehensive best practice guidelines for the management of HIAP in patients with previously undiagnosed FCS. In 2018, an expert panel first formulated dietary guidelines for FCS, with recommendations relating to acute pancreatitis that focused mainly on early aggressive IV hydration and emergent cessation of oral fat intake before institution of low‐LCT enteral nutrition containing MCTs.^19^ The authors recommended reducing daily fat intake to less than 10%–15% of total caloric intake, although this is difficult to sustain long‐term.^20^, ^21^ Three recent retrospective single‐centre studies presented long‐term dietary outcomes in small cohorts of patients with LPL deficiency.^20^, ^21^, ^22^ Thajer et al.^20^ supported a restriction of the daily dietary fat fraction to 10%–26% of total caloric‐intake as an effective and achievable target. Only 1/4 patients had pancreatitis on presentation, the acute management of which was not discussed. Kuthiroly et al.^22^ described a cohort of 15 FCS‐affected individuals with TG > 11.3 mmol/L on presentation. Only 1/15 presented with acute pancreatitis that was managed with IV rehydration and pain control for 48 h before initiation of a LCT fat‐restricted diet (<10% of total energy) supplemented with MCT.^23^ Finally, Aljouda et al.^21^ reported outcomes in six paediatric patients with LPL deficiency, advocating for long‐term management with an LCT‐restricted/MCT‐enriched diet. One individual had acute pancreatitis on presentation, managed with plasmapheresis until normalisation of TGs prior to commencing dietary fat restriction.

The two patients reported here were managed consecutively, with learnings from the first case informing management of the second. The dietary interventions were in accordance with recommendations for the early establishment of an LCT‐restricted/MCT‐enriched diet after a limited period of IV hydration (Table 2).^19^ Breast milk and standard infant formulas contain large amounts of LCT and are not suitable.^19^ Unlike LCT, MCTs, including caprylic acid (C8) and capric acid (C10), are not incorporated into chylomicrons but are instead hydrolyzed and bound to albumin for travel via the portal vein to the liver where they are oxidised to ketones.^19^ MCT supplementation is thus favoured to optimise caloric intake and macronutrient composition, without increasing circulating TG levels.^24^ The diet must also ensure adequate provision of EFA (~2%–4% of daily calories) and fat‐soluble vitamins and minerals.^19^ Carbohydrate intake should also be limited to 60% of daily caloric intake, as excess carbohydrates can promote increased production of TG‐rich VLDLs.^19^

Early management challenges for Patient 1 included a sudden rise in TGs (205 mmol/L) and lipase (2636 U/L) on day 2.5 of admission, after an initial period of improvement (Figure 1B). We hypothesise that this may have occurred in the context of a persistent catabolic state, exacerbated by inadequate caloric supplementation and rapid weaning of the insulin infusion. Low albumin levels on admission may have also impacted MCT absorption.^24^ Specialist metabolic dietitian input was critical to implementing a suitable modular feed that met nutritional requirements and promoted anabolism, ensuring an average daily caloric intake of 110–120 kcal/kg/day (103%–112% EER). This strategy of caloric optimisation also proved pivotal in the management of Patient 2 (Table 2).

Patient 1 had a more severe presentation requiring admission to intensive care. Heparin and insulin were initiated early in the management of the patient's HIAP, but therapeutic plasmapheresis was not used. While the potential role for plasmapheresis in the treatment of HIAP in FCS has only been suggested in isolated case reports,^25^, ^26^ recent American Society of Apheresis Guidelines point to the lack of high‐quality evidence to support this (category III, grade 2C).^27^ Therapeutic plasma exchange may lower TGs faster than cessation of oral intake. However, observational studies conducted in adults with multifactorial chylomicronemia syndrome (MCS) presenting with severe HTG and pancreatitis, showed no short‐term or long‐term benefit in those who did or did not receive plasmapheresis.^28^ Heparin and insulin have been used in HIAP without clear evidence of benefit and mostly based on case reports or small case series.^2^ Heparin releases heparan sulphate–LPL complexes from the capillary endothelium, which in patients harbouring pathogenic missense variants in *LPL* may potentially facilitate the enzyme's residual hydrolytic activity toward chylomicrons.^29^, ^30^, ^31^ Its use, however, needs to be balanced against the potential increased risk of bleeding. Insulin promotes synthesis and activation of LPL in muscle and adipose tissue, so its beneficial effect in children with LPL deficiency is thought to occur through stimulation of residual enzyme activity.^30^, ^32^ Insulin may be used in adult MCS patients with pancreatitis due to poorly controlled Type 2 diabetes or in patients with Type 1 diabetes with diabetic ketoacidosis in whom hypertriglyceridemia is not uncommon.^30^, ^33^ While some have suggested broadening its use to include normoglycemic patients with MCS, this requires co‐administration with glucose and careful monitoring for potential hypoglycaemia. More evidence is needed to determine its efficacy in LPL deficient patients, however.^2^ Interestingly, Ayyavoo et al.^32^ reported on a 71‐day‐old infant with LPL deficiency and severe recurrent HIAP who was acutely managed with an insulin‐dextrose infusion, followed by dietary fat restriction and high caloric intake (150 calories/kg/day). Overall, however, the evidence for the use of insulin, heparin or plasmapheresis in FCS remains largely anecdotal and their use should thus be considered with caution.

### 4.5 Novel and emerging pharmacological therapies

Long‐term adherence to a fat‐restricted diet is challenging, underpinning the need for pharmacological therapies in FCS. Traditional lipid lowering medications, such as fibrates, niacin, and statins are minimally effective since these patients lack lipolytic capacity.^1^ New therapies targeting the LPL inhibitors, ApoC‐III and ANGPTL3, have recently been trialled in adults with FCS and homozygous familial hypercholesterolemia, respectively.^18^ Volanesorsen and Olezarsen are antisense oligonucleotide therapies that selectively impair translation of ApoC‐III mRNA.^34^, ^35^ In separate randomised, placebo‐controlled trials involving adults with FCS, Volanesorsen and Olezarsen both significantly reduced triglyceride levels compared to placebo.^35^, ^36^ Furthermore, a recent meta‐analysis reported a reduction in the incidence of acute pancreatitis among patients with severe hypertriglyceridemia treated with Volanesorsen.^37^ Volanesorsen has received conditional marketing authorization by the European Medicines Agency for use in adult patients with FCS in whom response to diet and TG‐lowering therapy has been inadequate (https://www.ema.europa.eu/en/documents/product‐information/waylivra‐epar‐product‐information_en.pdf). Encouragingly, a recent observational open‐label n‐of‐1 study conducted in a paediatric patient harbouring null/null variants in LPL also showed that Volanesorsen effectively lowered TG levels by>70% and prevented hospital admissions for pancreatitis.^38^ More research is needed to establish appropriate dosing regimens of this drug for paediatric patients with LPL deficiency.^38^

## 5 CONCLUSION

FCS is a rare condition that can present with acute pancreatitis in infants and children. High index of suspicion, early diagnosis and appropriate dietary management with an LCT‐restricted/MCT‐enriched diet are important to avoid short and long‐term complications. Evidence‐based clinical guidelines are needed to guide stabilisation of infants with newly diagnosed FCS who present with HIAP. Beyond the currently available recommendations, this report also emphasises the importance of reversing catabolism through optimal caloric supplementation and consideration of heparin and insulin infusions for more severely affected patients.

## AUTHOR CONTRIBUTIONS

Clinical patient care and diagnosis: **O. H.**, **B. A.**, **E. G. B.**, **N. J. B.**, **M. S. E.**, **S. K.**, **T. H. R.**, **J. Y. L**. Evaluation of metabolic, genetic, and radiologic results: **O. H.**, **B. A.**, **J. S.**, **L. S.**, **E. S.**, **E. G. B.**, **N. J. B.**, **M. S. E.**, **S. K.**, **T. H. R.**, and **J. Y. L**. Planning of the manuscript: **O. H.**, **B. A**. Drafting of the manuscript: **O. H.**, **B. A**. Revision of the manuscript: **O. H.**, **B. A.**, **J. S.**, **L. S.**, **E. S.**, **E. G. B.**, **N. J. B.**, **M. S. E.**, **S. K.**, **T. H. R.**, **J. Y. L**.

## CONFLICT OF INTEREST STATEMENT

Oliver Heath, Brooke Allender, Joel Smith, Lucy Spencer, Elena Savva, Thomas H. Rozen, Natasha J. Brown, Elizabeth G. Bannister, Maureen S. Evans, Sharmila Kiss, and Joy Yaplito‐Lee have approved the manuscript and declare that they have no conflict of interest. They did not receive reimbursements/fees/funds/salaries from an organisation that may in any way gain or lose financially from the results reported in the reviewed manuscript in the last 5 years and have no other competing financial or non‐financial interests, as outlined in the JIMD Conflict of Interest form.

## INFORMED CONSENT

All procedures followed were in accordance with the Helsinki Declaration of 1975, as revised in 2000. Written informed consent was obtained from the patients’ parents for collection of samples and publication of medical data.

## ANIMAL RIGHTS

This article does not contain any studies with animal subjects performed by any of the authors.

## Supporting information


**APPENDIX S1:** Supporting information.

## Data Availability

This manuscript has no associated data.
