# Supplementary material for: Diagnosis and stabilisation of familial chylomicronemia syndrome in two infants presenting with hypertriglyceridemia‐induced acute pancreatitis
Source: JIMD Rep. 2024 Jun 2;65(4):239–48. doi: 10.1002/jmd2.12434 (PMC11224501; doi:10.1002/jmd2.12434)
Supplement: Supplementary file 1 — APPENDIX S1: Supporting information. [file JMD2-65-239-s001.pdf]

SUPPLEMENTARY MATERIALS

PLASMA LIPID ANALYSIS

All lipid analysis was performed on an Ortho Clinical Diagnostics Vitros XT7600 analyser in the Royal Children's Hospital Clinical biochemistry laboratory, accredited to ISO15189 standards. Triglycerides were measured using an enzymatic (lipase/glycerolkinase) method (reference range [RR] < 2 mml/L). HDL cholesterol was measured using a selective precipitation/cholesterol oxidase method and total cholesterol was measured with a cholesterol oxidase method (RR 2.5-4.9 mmol/L). LDL cholesterol was calculated from the measured lipid parameters using the Friedwald equation. Lipoprotein electrophoresis was performed using agarose gel electrophoresis with fat red 7B staining (Helena Laboratories) at NSW health pathology, Royal Prince Alfred Hospital.

GENOMIC ANALYSES

Patient 1

Trio whole genome sequencing (tWGS) was performed on DNA isolated from blood using massively parallel sequencing (NexteraTM DNA Flex Library Prep kit, Illumina Sequencers) with a mean target coverage of 30x and a minimum of 90% of bases sequenced to at least 10x for nuclear DNA (nDNA) and a minimum of 800x mean coverage for mitochondrial DNA (mtDNA). Data were processed, including read alignment to the reference genome (GRCh38) and to the revised Cambridge Reference Sequence (rCRS) mitochondrial genome (NC_012920.1). Variant calling was carried out using Cpipe or Mutect2 for nDNA and mtDNA, respectively.[1] For nDNA, variant analysis and interpretation within the target region (RefSeq genes +/-1Kb) was performed using Agilent Alissa Interpret and reported in accordance with HGVS nomenclature. Copy number

variants were screened for using an internal detection tool, CxGo.[2] Curation of nDNA variants was phenotype-driven with custom gene and pre-curated gene lists (https://panelapp.agha.umccr.org/) used for variant prioritisation: A*POC2, GPD1, LPL, APOA5, GPIHBP1, LMF1*, Pancreatitis v1.3, Red cell disorders v1.17, Dyslipidaemia v0.34, Familial hypercholesterolaemia v0.25, Miscellaneous Metabolic Disorders v1.20, Mendeliome v1.159, All genes excl Incidentalome v38.25.1.2. Variant classification was based on ACMG guidelines.[3]

Patient 2

Patient 2, trio whole exome sequencing (tWES) was performed on DNA isolated from blood using massively parallel sequencing (Twist VCGS whole exome capture, Illumina Sequencers) with a minimum coverage of 97% of bases sequenced to at least 10x. Data processing, including read alignment to the reference genome (GRCh38) and variant calling, was carried out using Cpipe.[1] Variant analysis and interpretation within the target region (coding exons +/-8bp) was performed using Agilent Alissa Interpret. Curation of variants was phenotype-driven with pre-curated gene lists (https://panelapp.agha.umccr.org/) used for variant prioritisation: Dyslipidaemia v0.34, Familial hypercholesterolaemia v0.25, Pancreatitis v 1.3. Variant classification was based on ACMG guidelines.[3]

REFERENCES

1 Sadedin, S. P. *et al.* Cpipe: a shared variant detection pipeline designed for diagnostic settings. *Genome Med* **7**, 68, doi:10.1186/s13073-015-0191-x (2015).

2 Sadedin, S. P., Ellis, J. A., Masters, S. L. & Oshlack, A. Ximmer: a system for improving accuracy and consistency of CNV calling from exome data. *Gigascience* **7**, doi:10.1093/gigascience/giy112 (2018).

3 Richards, S. *et al.* Standards and guidelines for the interpretation of sequence variants: a joint consensus recommendation of the American College of Medical Genetics and Genomics and the Association for Molecular Pathology. *Genet Med* **17**, 405-424, doi:10.1038/gim.2015.30 (2015).
